# DNA hypermethylation in the normal colonic mucosa of patients with colorectal cancer

**DOI:** 10.1038/sj.bjc.6602940

**Published:** 2006-01-17

**Authors:** K Kawakami, A Ruszkiewicz, G Bennett, J Moore, F Grieu, G Watanabe, B Iacopetta

**Affiliations:** 1 Department of Surgery, Kanazawa University School of Medicine, Takaramachi 13-1, Kanazawa 920-8641, Japan; 2 Divisions of Tissue Pathology, Institute of Medical and Veterinary Science, Frome Road, Adelaide SA 5000, Australia; 3 Molecular Pathology, Institute of Medical and Veterinary Science, Frome Road, Adelaide SA 5000, Australia; 4 Colorectal Unit, Royal Adelaide Hospital, Adelaide SA 5000, Australia; 5 School of Surgery and Pathology, University of Western Australia, Nedlands 6009, Australia

**Keywords:** *ERα*, *MYOD*, promoter methylation, *DNMT3b*, polymorphism, ageing

## Abstract

The CpG-island methylator phenotype (CIMP+) in colorectal cancer (CRC) is characterised by frequent hypermethylation of promoter regions in tumour suppressor genes. Low level methylation of some CpG islands is also seen in the normal colonic mucosa and increases with age; however, it is still unclear what other factors regulate this phenomenon. The first aim of our study was to determine whether the level of promoter methylation is elevated in the normal colonic mucosa of patients with CIMP^+^ tumours. The second aim was to investigate whether common, functional polymorphisms in genes involved in methyl group metabolism are associated with the level of methylation in this tissue. CpG islands within the *ERα*, *MYOD*, *P16(INK4A)*, *MLH1*, *APC*, *P14(ARF)*, *DAPK* and *TIMP3* genes were quantitatively evaluated for methylation in normal colonic mucosa from a large series of CRC patients using the MethyLight assay. Genotyping was carried out for polymorphisms in the *MTHFR*, *TS*, *MS*, *MTHFD1* and *DNMT3b* genes. Methylation of *ERα* and *MYOD* in normal colonic mucosa increased with age and was higher in female subjects. Methylation of *P16(INK4A)*, *MLH1*, *TIMP3* and *DAPK* in normal mucosa occurred at a lower level than *ERα* and *MYOD* but also increased with age and was significantly higher in patients with CIMP^+^ tumours. The *DNMT3b* C46359T polymorphism was associated with significantly less methylation of *MYOD* and *MLH1* and with trends for lower methylation in each of the other CpG islands examined. Our results demonstrate that age, gender and genetic factors can influence the methylation level of CpG islands in gene promoter regions of normal colonic mucosa. Further work is required to determine whether such methylation is associated with the development of CIMP^+^ CRC.

Changes in DNA methylation patterns are frequently observed in neoplastic cells (Baylin *et al*, 1998). Both global hypomethylation (Feinberg and Vogelstein, 1983) and CpG-island hypermethylation (Toyota *et al*, 1999) occur simultaneously in colorectal cancer (CRC); however, there appears to be no relationship between these epigenetic alterations (Bariol *et al*, 2003). Hypermethylation of CpG islands is associated with transcriptional silencing of tumour suppressor genes including *P16(INK4A)* (Merlo *et al*, 1995) and *MLH1* (Kane *et al*, 1997). The term CpG-island methylator phenotype (CIMP^+^) was proposed several years ago to describe tumours that display frequent and concurrent hypermethylation of multiple CpG islands (Toyota *et al*, 1999). CIMP^+^ CRCs show characteristic clinical and pathological features that include origin in the proximal colon, higher frequency in female subjects and poorly differentiated, mucinous histology (Toyota *et al*, 1999; Hawkins *et al*, 2002; Van Rijnsoever *et al*, 2002). Whether these properties arise because of aberrant DNA hypermethylation or as a consequence of the microsatellite instability (MSI^+^) phenotype found in a high proportion of CIMP^+^ tumours following methylation-induced transcriptional silencing of *MLH1* has been highly contentious (Yamashita *et al*, 2003; Frigola *et al*, 2005). However, recent evidence suggests that many of the characteristic features of CIMP^+^ tumours including poor differentiation, mucinous histology, proximal site and *BRAF* mutation are found independently of MSI^+^ status (Whitehall *et al*, 2002; Kambara *et al*, 2004; Samowitz *et al*, 2005a, 2005b). The CIMP^+^/MSI^−^ tumour subgroup has worse prognosis (Ward *et al*, 2003; Samowitz *et al*, 2005b), but the overall CIMP^+^ phenotype may be more responsive to 5-fluorouracil-based chemotherapy (Van Rijnsoever *et al*, 2003).

The methylation of some CpG islands in the normal colonic mucosa has been shown to increase with age (Issa *et al*, 1994; Ahuja *et al*, 1998; Nakagawa *et al*, 2001). This has led to a proposal by Toyota *et al* (1999) that methylation of some genes in this tissue is age-related (Type A genes), whereas for other genes the methylation is cancer-specific (Type C genes). Examples of Type A genes include *ERα* and *MYOD* and for Type C genes they include *P16(INK4A)*, *MLH1* and *TIMP3*. This classification could be misleading, however, in that age-related differences in methylation levels may be quantitative rather than qualitative. Age-related methylation and subsequent inactivation of tumour suppressor genes has been suggested as a predisposing factor for the increased risk of cancer with age (Issa, 1999).

The factors that regulate both global DNA methylation and site-specific hypermethylation in normal and neoplastic tissues are largely unknown. Age clearly has some influence, with dietary folate intake, alcohol consumption and gender also likely to be important factors (Choi and Mason, 2002; Kim, 2004). Genetic factors could also play a role in determining the level of normal tissue DNA methylation and hence the risk of cancer. Common, functional polymorphisms have been described for several genes involved in methyl group metabolism. Most attention has so far focused on variants of the methylenetetrahydrofolate reductase (*MTHFR*), thymidylate synthase (*TS*), methionine synthase (*MS*), DNA methyltransferase (*DNMT3b*) and methylenetetrahydrofolate dehydrogenase (*MTHFD1*) genes (Friso *et al*, 2002; Paz *et al*, 2002; Kawakami *et al*, 2003; Chen *et al*, 2004; Sharp and Little, 2004). We previously reported the wild-type *MTHFR* 677C allele to be associated with significantly higher concentrations of two important intracellular folate intermediates compared to 677T homozygous variants in CRC (Kawakami *et al*, 2003). These folate intermediates were present at almost twice the concentration in CIMP^+^ compared to CIMP^−^ tumours, suggesting that the *MTHFR* C677T polymorphism could also influence the level of DNA hypermethylation by altering the intracellular methyl donor pool. Work by other groups has implicated this polymorphism in the level of global DNA methylation in normal tissues (Stern *et al*, 2000; Friso *et al*, 2002; Paz *et al*, 2002).

In the present study, we used a quantitative assay to evaluate the methylation level in DNA from normal colonic tissue of eight CpG islands located within gene promoter regions. We were thus able to investigate for possible influences of age, gender and methyl group-related genetic factors on the level of CpG-island methylation. The finding of strong associations between high levels of normal colonic tissue methylation and the presence of CIMP^+^ tumours suggests that there may be a causal link.

## MATERIALS AND METHODS

### Tissue samples

Tissue samples from a consecutive series of 199 CRC patients undergoing elective surgery at the Colorectal Unit of the Royal Adelaide Hospital were snap frozen in liquid nitrogen within 20–40 min after resection and stored at −70°C. Patients had been fasted for 24 h prior to surgery. Samples of normal mucosa were taken as far away as possible from the tumour site and were morphologically normal in sections used to evaluate the status of that margin in routine histology. The normal sample was usually the distal resection margin for proximal colon cancers or the proximal resection margin for left colon and rectal cancers. Microsatellite instability status was determined as previously described by screening for instability at nine microsatellite loci that included both mononucleotide (BAT-25, BAT-26, BAT-40) and dinucleotide (D2S123, D10S197, D17S579, D18S34, D5S346, D17S250) repeats (Ruszkiewicz *et al*, 2002). Tumours showing instability at two or more loci were considered to be MSI^+^. Tumours showing methylation at two or more of six CpG islands examined (*P16(INK4A)*, *MLH1*, *APC*, *TIMP3*, *P14(ARF)*, *DAPK*) were classified as CIMP^+^ (Kawakami *et al*, 2003). Information on patient age, gender and tumour characteristics (stage, nodal involvement, grade, mucinous histology, infiltrating lymphocytes) were obtained from the pathology report. Ethics approval for this study was received from the Sir Charles Gairdner Hospital Human Research Ethics Committee.

### Methylation analysis

Aliquots of normal mucosa and tumour tissue were sent on dry ice to the Kanazawa University School of Medicine for extraction of DNA and quantitative assessment of CpG-island methylation within the promoter regions of *ERα*, *MYOD*, *MLH1* (distal promoter region), *P16(INK4A)*, *TIMP3*, *P14(ARF)*, *APC* and *DAPK*. This was carried out using the MethyLight assay and oligonucleotide primer sequences described previously (Eads *et al*, 2001; Ishiguro *et al*, 2003; Kawakami *et al*, 2003). Briefly, tumour DNA was converted with sodium bisulphite prior to analysis of methylation using the fluorescence-based, real-time PCR MethyLight assay. The results were analysed to obtain a percentage of methylated reference (PMR) value as described previously (Eads *et al*, 2001). *ERα*, *MYOD*, *TIMP3* and *DAPK* were evaluated for methylation in 188 normal mucosal samples; *MLH1*, *P16(INK4A)*, *p14*(*ARF)* and *APC* in 100 samples. These were categorised into no methylation (PMR=0), low methylation (PMR⩽median value of positive readings) and high methylation (PMR>median value of positive readings).

### Genotyping

PCR and PCR-RFLP were used to genotype the 28 bp tandem repeat in the enhancer region of *TS* and the G/C single-nucleotide polymorphism (SNP) within the triple repeat allele, respectively (Kawakami and Watanabe, 2003). The *Hae*III-digested PCR product sizes were estimated using 3% agarose gels. Genotyping for the *MTHFR* C677T and A1298C SNPs was carried out using PCR-F-SSCP as described earlier by our laboratory (Grieu *et al*, 2004). PCR-RFLP was used to genotype for SNPs in the *MTHFD1* (G1958A), *MS* (A919G) and *DNMT3b* (C46359T) genes using the restriction enzymes *Msp*I (Promega, Sydney), *Hae*III (Promega) and *Avr*II (New England BioLabs, Brisbane), respectively, as described earlier (Paz *et al*, 2002; Shen *et al*, 2002; Chen *et al*, 2004).

### Statistical analysis

Statistical analyses were performed using the SPSS software package (Chicago, IL, USA). As methylation levels are not normally distributed, median methylation levels between different groups were compared using the Mann–Whitney *U*-test, with PMR values treated as continuous variables. Associations between age and methylation were evaluated using linear regression, while associations between methylation levels (categorised as none, low or high as described above) and clinical, pathological and genotypic features were evaluated using *χ*^2^ and Fishers' exact test as appropriate. All *P*-values given are two tailed with *P*<0.05 taken as statistically significant.

## RESULTS

The MethyLight assay (Eads *et al*, 2001) was used to obtain quantitative estimates for the level of methylation of CpG islands within the *ERα*, *MYOD*, *P16(INK4A)*, *MLH1*, *APC*, *P14*(*ARF)*, *DAPK* and *TIMP3* genes in normal colonic mucosa from patients with CRC. Consistent with previous reports (Toyota *et al*, 1999; Rashid and Issa, 2004), *ERα* and *MYOD* showed high methylation levels in this tissue (Table 1). *P16(INK4A)*, *MLH1*, *APC*, *DAPK* and *TIMP3* each showed relatively low methylation levels in normal colonic tissue (mean PMR<1), while *P14(ARF)* methylation could not be detected using the MethyLight assay.

**Figure 1:**
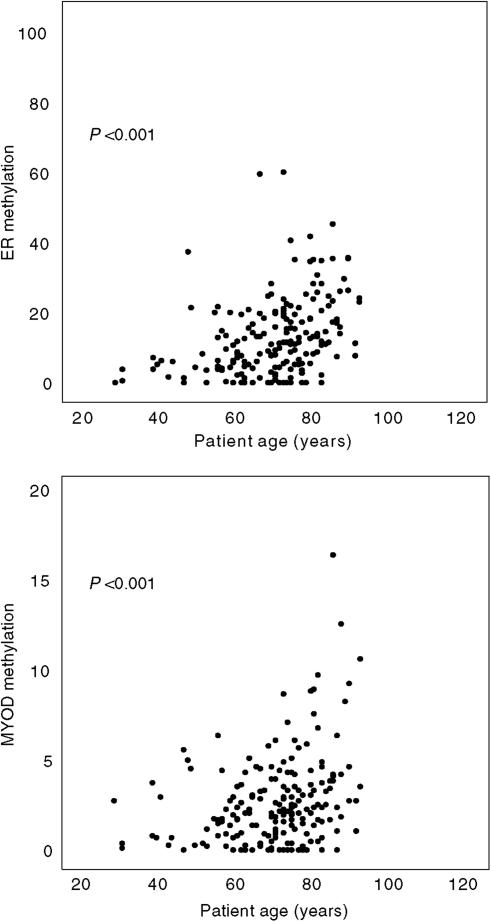
Methylation levels for *ERα* and *MYOD* in the normal mucosa of CRC patients in relation to age at diagnosis. Values were determined by MethyLight assay and are expressed as the percentage of a methylated reference (PMR). Older age was associated with significantly increased methylation of *ERα* and *MYOD* (*P*<0.001).

**Figure 2:**
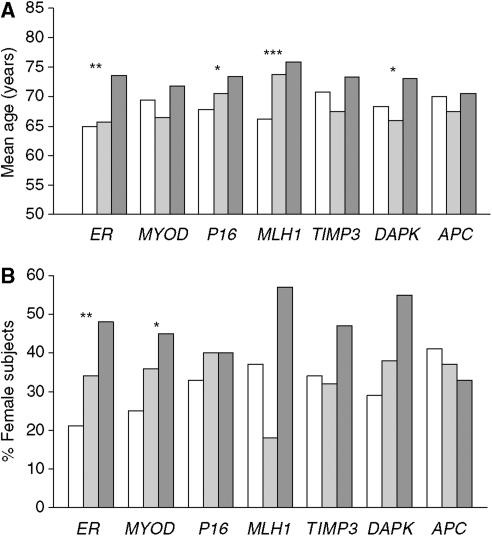
Methylation levels in normal colonic tissue were categorised according to no detectable methylation (PMR=0; open bars), low methylation (PMR ⩽median of positive values; light bars) and high methylation (PMR >median of positive values; dark bars). A high level of methylation in normal colon tissue was associated with older age (**A**) and female gender (**B**) compared to no detectable methylation. ^*^P<0.1; ^**^P<0.05; ^***^P<0.01.

*ERα* and *MYOD* methylation in normal colonic tissues increased significantly with patient age (*P*<0.001 for each; Figure 1). When methylation levels were categorised into none, low or high, patients with high *ERα* and *MLH1* methylation were found to be significantly older than those with no methylation of these genes (Figure 2A). Patients with high *P16(INK4A)* and *DAPK* methylation also showed trends for older age. Significantly, more female subjects showed high levels of *ERα* methylation relative to the no methylation group (Figure 2B), with *MYOD* methylation showing the same trend. Almost half of the patients with high *ERα* methylation were female subjects compared to only 21% of those with no methylation of this gene. No associations were seen between the methylation level of any gene investigated and anatomical site of the normal mucosa in the large bowel (results not shown).

**Figure 3:**
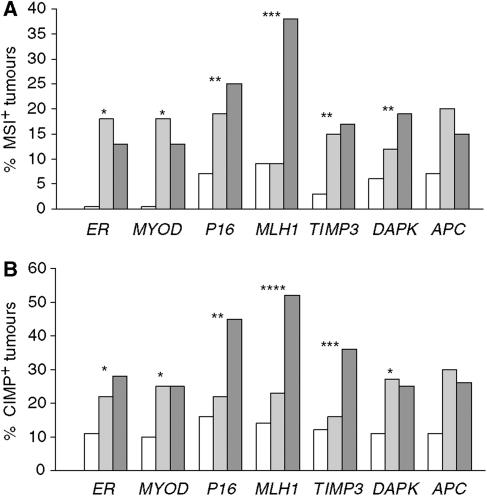
High methylation levels in normal colon tissue were more often associated with the presence of MSI^+^ (**A**) and CIMP^+^ (**B**) tumours compared to cases with no detectable methylation. ^*^P<0.1; ^**^P<0.05; ^***^P<0.01; ^****^P<0.001. No detectable methylation (open bars), low methylation (light bars) and high methylation (dark bars) groups are shown.

Normal colonic tissue from patients with MSI^+^ and CIMP^+^ tumours frequently demonstrated high methylation levels, particularly for the *P16*(*INK4A*), *MLH1*, *TIMP3* and *DAPK* genes (Figure 3). High methylation levels of *MLH1* were seen in the normal tissue of more than one-third of patients with MSI^+^ tumours and just over half the patients with CIMP^+^ tumours. Similar trends were also apparent for *ERα*, *MYOD* and *APC*. In contrast, the absence of detectable methylation for each of the genes examined in normal mucosa was observed in <10% of patients with MSI^+^ tumours and in <20% of patients with CIMP^+^ tumours. Of the 44 CIMP^+^ tumours in this cohort, 28 (64%) were MSI^−^ and 16 (36%) were MSI^+^, thus allowing us to examine the CIMP^+^/MSI^−^ tumour group separately to the CIMP^+^/MSI^+^ group. Although the sample sizes were small, we observed higher *MLH1* methylation (*P*=0.05) but lower *ERα* methylation (*P*=0.025) in the normal mucosa of patients with CIMP^+^/MSI^+^ tumours compared to those with CIMP^+^/MSI^−^ tumours. None of the other genes showed significant differences in methylation level between the two groups.

**Figure 4:**
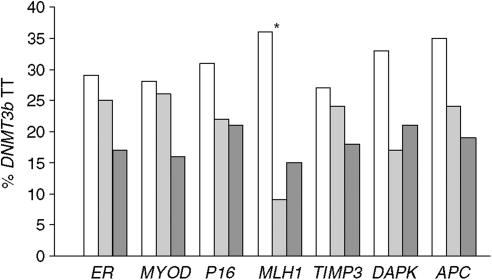
Colorectal cancer patients with no detectable methylation in normal colon tissue were more often homozygous for the *DNMT3b* C46359T variant compared to patients with high levels of methylation. Although not reaching statistical significance, this trend was seen for all seven genes investigated. No detectable methylation (open bars), low methylation (light bars) and high methylation (dark bars) groups are shown. ^*^P<0.1.

We next investigated whether common polymorphisms in the methyl group metabolism genes *MS*, *MTHFR*, *MTHFD1*, *DNMT3b* and *TS* were associated with the levels of CpG-island methylation in normal colonic tissue. Only the *DNMT3b* C46359T variant was consistently associated with altered methylation levels in normal colonic mucosa. Methylation levels of *MYOD* and *MLH1* were both significantly lower in *DNMT3b* TT homozygous patients compared to the combined CC/CT group of patients (Mann–Whitney *U*-test, *P*=0.027 and *P*=0.013, respectively), with methylation in each of the other genes showing the same trend. For each gene, the highest percentage of *DNMT3b* TT homozygous genotype was seen in patients who had no detectable methylation in their normal colonic mucosa (Figure 4).

## DISCUSSION

Type A methylation sites were originally proposed to describe CpG islands that are methylated in normal tissue and whose methylation increases with age, with the best-known examples being *ERα* and *MYOD* (Toyota *et al*, 1999). In contrast, Type C CpG islands were proposed as being methylated exclusively in tumour tissue, with *P16(INK4A)* and *MLH1* suggested as examples. In the present study, we used a quantitative assay to evaluate DNA methylation levels in normal colonic tissue from CRC patients who underwent surgical treatment. As expected, methylation levels for *ERα* and *MYOD* in normal colonic mucosa were higher than the other genes evaluated (Table 1) and increased with age (Figure 1). However, we obtained clear evidence that methylation levels for *P16(INK4A)*, *MLH1* and *DAPK* also increased with age in the normal colonic mucosa (Figure 2A). Moreover, although the strongest associations between high methylation levels and female gender were observed for *ERα* and *MYOD*, a similar trend was apparent for all other genes investigated with the exception of *APC* (Figure 2B). These results argue against a simple classification of CpG islands into Type A or Type C and suggest instead that differences in their methylation levels in normal colonic tissue are quantitative rather than qualitative.

The second major finding of this study was that promoter methylation levels in normal colonic mucosa showed no apparent associations with site in the large bowel. This was an unexpected observation because tumour methylation levels for most of the genes examined here are known to be much higher in cancers arising in the proximal colon compared to those from the distal colon or rectum (Toyota *et al*, 1999; Hawkins *et al*, 2002; Van Rijnsoever *et al*, 2002). We caution, however, that additional studies involving more precise sampling of normal colonic mucosa from individuals without cancer are required before concluding that there are no site-related differences in the CpG-island methylation levels of this tissue.

The third major finding of the current study was the presence of relatively high methylation levels in the normal colonic tissues of patients with MSI^+^ and CIMP^+^ tumours (Figure 3). This was particularly apparent for *P16(INK4A)*, *MLH1*, *TIMP3* and *DAPK* but was seen for all genes examined. Elevated *MLH1* methylation has previously been reported in the normal gastric epithelia of patients with stomach cancer compared to noncancer individuals (Waki *et al*, 2002). Increased *MGMT* methylation in the normal mucosa of patients with colon cancers showing *MGMT* methylation was also reported recently (Shen *et al*, 2005). Together, these observations suggest that methylation levels in the normal colonic mucosa could serve as markers of risk for the development of CRC and in particular for the closely related MSI^+^/CIMP^+^ subgroup. Longitudinal studies performed on individuals at high risk of developing CIMP^+^ CRC will be required to test this hypothesis.

Older age and female gender (Figures 1 and 2) are generally associated with higher methylation levels for most CpG islands in normal colonic mucosa. However, it is clear that considerable interindividual variation occurs in the methylation level for this tissue, with some young patients showing relatively high values (Figure 1). Genetic, dietary and environmental factors are therefore also likely to be important in determining CpG-island methylation levels. Dietary information was not available for this patient series and hence could not be investigated. We did, however, investigate several common, functional polymorphisms in genes known to play major roles in methyl group metabolism, with only the *DNMT3b* C46359T variant found to be associated with methylation levels in normal mucosa (Figure 4). Although the sample size was relatively small, all seven CpG islands investigated in this study showed less methylation in *DNMT3b* TT homozygous individuals compared to those with the CC/CT genotype. *DNMT3b* mediates *de novo* DNA methylation, with the C/T transition polymorphism postulated to increase *in vitro* transcriptional activity by 30% (Shen *et al*, 2002). The lower methylation level observed here for *DNMT3b* TT homozygotes is therefore contrary to expectations and this gene could influence the level of global DNA methylation independently of the hypermethylation of CpG islands in promoter regions. Additional studies performed on large cohorts of noncancer individuals and incorporating dietary information are required to confirm the novel association reported here between *DNMT3b* genotype and CpG-island methylation levels in normal colonic mucosa.

Although the *DNMT3b* C46359T variant appears to be associated with CpG-island methylation levels in the normal colonic mucosa (Figure 4), we have not determined whether this polymorphism also confers a risk for the development of CIMP^+^ CRC. The contribution of genetic factors to the development of CIMP^+^ is controversial, with some workers reporting an association between family history of CRC and CIMP^+^ (Frazier *et al*, 2003; Young *et al*, 2005), but not others (Ward *et al*, 2004). Functional polymorphisms in methyl group metabolism and DNA methylation genes are clearly interesting candidates for further study and are currently being investigated in large cohorts of well-defined CIMP^+^ tumours.

In conclusion, methylation levels for most CpG islands in normal colonic mucosa increase with age and are higher in female subjects. Unlike colorectal tumours, methylation levels in the normal mucosa do not vary according to anatomical site in the large bowel. In addition to age and gender, a common genetic variant in the DNA methyltransferase gene *DNMT3b* also appears to influence CpG-island methylation levels in normal colonic mucosa. The methylation levels of several genes in normal colonic mucosa were associated with the presence of MSI^+^ and CIMP^+^ tumours and could thus serve as molecular-based markers of risk for the development of this CRC subgroup.

## Figures and Tables

**Table 1:** DNA methylation level in the normal colonic mucosa of CRC patients

**CpG-island (*N*)**	**Mean PMR (range)**	**Median positive PMR**
ER*α* (188)	13.3 (0–99.1)	12.3
*MYOD* (188)	2.8 (0–19.3)	2.4
P16(INK4A) (100)	0.14 (0–1.1)	0.2
*MLH1* (100)	0.17 (0–0.9)	0.3
*APC* (100)	0.47 (0–5.3)	0.5
*DAPK* (188)	0.67 (0–4.5)	0.6
*TIMP3* (188)	0.78 (0–9.5)	0.6
